# Prevalence and risk factors of depression among undergraduate medical students in a Nigerian university

**DOI:** 10.4314/gmj.v56i4.9

**Published:** 2022-12

**Authors:** Alphonsus R Isara, Ogechukwu I Nwokoye, Agatha O Odaman

**Affiliations:** Department of Community Health, University of Benin, Benin City, Nigeria

**Keywords:** Depression, risk factors, undergraduate medical students, University of Benin, Nigeria

## Abstract

**Objectives:**

This study assessed the prevalence and risk factors of depression among undergraduate medical students at the University of Benin, Benin City, Nigeria.

**Design:**

This was a cross-sectional study.

**Setting:**

This study was carried out at the University of Benin.

**Participants:**

Three hundred medical students were recruited for this study.

**Methods:**

The Patient Health Questionnaire 9 (PHQ-9) and a structured pre-tested self-administered questionnaire were used to assess the prevalence and risk factors of depression, respectively.

**Results:**

The students' age ranged from 15 – 34 years, with a mean age of 21.8 ± 3.3 years. Many risk factors which could predispose students to depression were identified. Overall, 96 (32.0%) students were found to have depression. Of these, 59 (19.0%) had mild depression, 4 (1.3%) had severe depression, 53 (39.3%) were pre-clinical students, and 43 (26.1%) were clinical students. Emotional problems (OR 2.205, 95% CI 1.122 – 3.749, p = 0.020), financial challenges (OR 3.971, 95% CI 2.170 – 7.269, p < 0.001) and smoking (OR 6.877, 95% CI 1.731 – 27.327, p = 0.006) were the significant independent predictors of depression.

**Conclusion:**

The prevalence of depression was high among medical students. There is a need to include screening for risk factors of depression in the routine medical examinations for new students admitted into medical schools.

**Funding:**

None declared

## Introduction

Globally, there is an increasing burden of depression and other mental health conditions. The World Health Organization (WHO) in January 2020 estimated that “more than 264 million people of all ages suffer from depression”.^1^ The disease usually manifests as feelings of sadness, loneliness and mental breakdown while retaining the ability to carry out daily activities and tasks in mild cases, to feelings of low self-esteem, self-blame, anger, peevishness, inability to perform daily activities and tasks, suicidal ideation and suicide in the worst of cases.^2^ Unfortunately, low and middle-income countries bear the brunt of depression as most people in these countries receive no treatment for it.^3^ In Nigeria, Gureje et al. reported “a lifetime prevalence rate of a major depressive episode of 3.1% among adults aged 18 years and above”.^4^ This translates to over six million people because the population of Nigeria is well over 200 million.

In recent years, there has been a steady increase in the number of undergraduate medical students in Nigeria. Because medical students spend long years in the university, and their training curriculum is different from that of other departments, however, exposure to the same social and environmental conditions predisposing to stress could result in mental disorders, including depression. A meta-analysis of 25 studies in Brazil revealed that 30.6% of medical students had depression.^5^ Studies in Cameroon and Ethiopia reported 30.6% and 51.3% prevalence among medical students.^6^,^7^ In Nigeria, several studies in the southeastern and southwestern parts of the country have reported a prevalence of severe depression ranging from 1.6% to 7% and mild depression ranging from 25.2% to 71.8% among undergraduate medical students.^8^–^10^ As in the general population, where many people suffering from depression do not perceive that they have a treatable illness, studies in different countries have shown poor help-seeking behaviour for depression among students.^9^,^11^

Depression has several negative effects on students, some of which includes: personal, cognitive, and emotional problems, notably, decision-making and problems of time management; poor academic achievement and low level of exam performance; decreased attention and drug abuse; overconsumption of alcohol and increased levels of smoking; and negative effects on everyday work and achievements.^13^,^14^ The most serious consequence of depression is the threat of suicide. It has been documented that depression is the most prevalent cause of suicide attempts among students.^5^,^14^–^18^

Several risk factors for depression have been identified among undergraduate medical students, just like in the general population. These factors include but not limited to the following: stress from academic activities; comorbidities like post-traumatic stress disorders (PTSD), hypertension, diabetes mellitus, anxiety and sleeping disorders; lack of social support; relationship problems; female gender, younger age group and financial constraints.^6^–^8^,^10^,^12^,^15^ The female undergraduate medical student population is increasing in Nigeria. This trend calls for specific actions because several studies have shown that being female is a very important predictor of depression.^19^–^21^ Stress is a major precursor of depression among students. This is corroborated by a study in eight medical schools across Nigeria, which reported that most of the students perceived the programme as very stressful.^22^

The burden of depression among undergraduate medical students calls for serious concern as medical students need to be psychologically sound and well prepared to function as medical doctors whose work is equally stressful, especially in the Nigerian context. Aguocha et al. reported a high prevalence of depression among resident doctors in a teaching hospital in South Eastern Nigeria’.^23^ The predisposing factors for depression may have been present in the doctors since their undergraduate days.

Given the nature of the academic curriculum in the medical school and the preponderance of socio-economic and environmental factors which could easily predispose adolescents and young people to depression in Nigeria, we hypothesize that there could be a huge burden of depression among medical students in the university. Thus, this study aimed to assess the prevalence and risk factors of depression among undergraduate medical students of the University of Benin, Benin City, Nigeria.

## Methods

### Study design, setting and population

This cross-sectional study was carried out from November 2019 to January 2020 among undergraduate medical students in the School of Medicine, University of Benin, Benin City, Nigeria.

The medical school was founded in 1970 and has a yearly admission of 150 students. In the first year (100 level), the students were exposed to science subjects in the Faculty of Science. At the second (200 level) and third year (300 level), referred to as the pre-clinical stage, they are exposed to the basic medical sciences of anatomy, physiology and biochemistry. The clinical stage from the fourth (400 level) to the sixth year (600 level) was spent on all the clinical courses with clinical orientation and rotations at the University of Benin Teaching Hospital. Only 200 to 600 level students were included in this study. Although the university has the Guidance and Counselling section in the Students' Affairs Department, there is no support system for students suffering from depression.

### Sample size calculation and sampling

The minimum sample size required for this study was calculated using the Cochran formula.^24^ The following assumptions were made: a proportion of 21.3% being the prevalence of depression across eight medical schools in Nigeria,^22^ 5% error margin, 95% confidence interval and 10% non-response rate. Thus, the calculated sample size was 287. Using the class list as a sampling frame, a systematic sampling technique was used to recruit students from various levels for the study. The sampling interval (k) was calculated by dividing the total number of students in the class (N) by the sample size allocated to that class (n). The first respondent in the class was selected by simple random sampling within the sampling interval and subsequently every nth student was recruited for the study until the sample size allocated to the respective class is reached. Proportional allocation was done to ascertain the number of students recruited from each academic level of study.

### Data collection

Data collection took place in the respective classrooms of the students across all levels of study. This was done either before commencement or at the end of lectures. Data on the prevalence of depression was collected using the validated Patient Health Questionnaire 9 (PHQ-9), which has a sensitivity of 88% and a specificity of 88%. The PHQ-9 is a self-administered version of the PRIME-MD diagnostic instrument for common mental disorders.^25^ It contains nine questions aimed at identifying depressive symptoms (first eight questions), including the presence and duration of suicide ideation (Question nine). Respondents were expected to tick one of four options, “Not at all”, “Several days”, “More than half the days”, and “Nearly every day” for the nine tabulated questions. The PHQ-9 scores are divided into increasing severity categories: 0-4, 5-9, 10-14, 15-19 and 20-27. These correspond to no depression, mild, moderate, severe, and severe depression, respectively.

A structured, pre-tested self-administered questionnaire was used to collect data on socio-demographic characteristics of the students and their prior exposure to risk factors for depression. This paper-based questionnaire was pre-tested among undergraduate medical students of a private University in Edo State and necessary corrections and adjustments were made to the questions. The questionnaire contained two sections. The first section sought information on the age, sex, marital status, study level and student's residence. The second section elicited exposure to various risk factors that could predispose the students to depression.

### Data analysis

Data analysis was done using IBM SPSS Statistics version 22.0 (IBM Corp, Armonk, NY, USA). The association between the independent variables (socio-demographics and risk factors of depression) and the prevalence of depression among the students was determined using the Chi-square test. Binary logistic regression was modelled to determine the significant predictors of depression. The level of statistical significance was set at p < 0.05.

### Ethical consideration

This study was approved by the University of Benin Teaching Hospital Ethics and Research Committee (protocol number: ADM/E 22/A/VOL. VII/148210). Written informed consent was obtained from the students, and they were assured of confidentiality before they were given the data collection tools. We ensured that the students completed the study instruments privately without interacting with other students, like in an examination. There were also no identifiers in the study instruments.

## Results

Three hundred undergraduate medical students with a mean age of 21.8 ± 3.3 years participated in the study. The age range of the students was 15 – 34 years. The highest proportion, 166 (55.3%) were aged 20-24. The male (47.0%) to female (53.0%) ratio was 1: 1.1. Most (96.7%) of the students were not married and were Christians. Students in the pre-clinical stage of their studies constituted 45.0% of the study population. The majority (73.0%) reside within the University campus, while a quarter (25.3%) of them live alone (Table 1).

**Table 1:** Socio-demographic characteristics of the medical students (n =300)

Variable	n (%)
**Age Group (years)** **15 – 19** **20 – 24** **25 – 30** **30 – 34**	81 (27.0) 166 (55.3) 43 (14.3) 10 (3.3)
***Mean ± Sd (21.8 ± 3.3)***	
**Sex** **Male** **Female**	141 (47.0) 159 (53.0)
**Marital Status** **Single** **Married**	290 (96.7) 10 (3.3)
**Religion** **Christianity** **Islam**	290 (96.7) 10 (3.3)
**Level of study** **Pre-clinical** **Clinical**	135 (45.0) 165 (55.0)
**Place of residence** **On campus** **Off campus**	219 (73.0) 81 (27.0)
**Reside alone** **Yes** **No**	76 (25.3) 224 (74.7)

The students' responses to questions of the PHQ-9 are shown in Table 2. For several days, one-third 101 (33.7%) of the students experienced little interest or pleasure in doing things, 76 (25.3%) had trouble falling or staying asleep or sleeping too much, 129 (43.0%) had a feeling of tiredness or having little energy, 80 (26.7%) had Poor appetite or overeating while 74 (24.7%) had trouble concentrating on things such as reading the newspaper or watching television. Also, 32 (10.7%) had tiredness or little energy nearly daily.

**Table 2:** The PHQ-9 responses of the medical students

How often were respondents bothered by any of the following problems two weeks before the study	Not at all n (%)	Several days n (%)	More than half the days n (%)	Nearly everyday n (%)
**Little interest or pleasure in doing things**	175 (57.3)	101 (33.7)	2 (0.7)	25 (8.3)
**Feeling down, depressed or hopeless.**	198 (66.0)	85 (28.3)	5 (1.7)	12 (4.0)
**Trouble falling or staying asleep or sleeping too much**	198 (66.0)	76 (25.3)	5 (1.7)	21 (7.0)
**Feeling tired or having little energy**	130 (43.3)	129 (43.0)	9 (3.0)	32 (10.7)
**Poor appetite or overeating**	191 (63.7)	80 (26.7)	11 (3.7)	18 (6.0)
**Feeling bad about yourself or that you are a** **failure or have let yourself or your family** **down**	228 (76.0)	58 (19.3)	4 (1.3)	10 (3.3)
**Trouble concentrating on things such as reading** **the newspaper or watching television**	207 (69.0)	74 (24.7)	4 (1.3)	15 (5.0)
**Moving or speaking so slowly that other people** **could have noticed? Or the opposite- being** **so fidgety or restless that you have been moving** **around a lot more than usual?**	245 (81.7)	43 (14.3)	0 (0.0)	12 (4.0)
**Thoughts that you would be better off dead or** **hurting yourself in some way**	265 (88.3)	22 (7.3)	1 (0.3)	12 (4.0)

**Figure 1:**
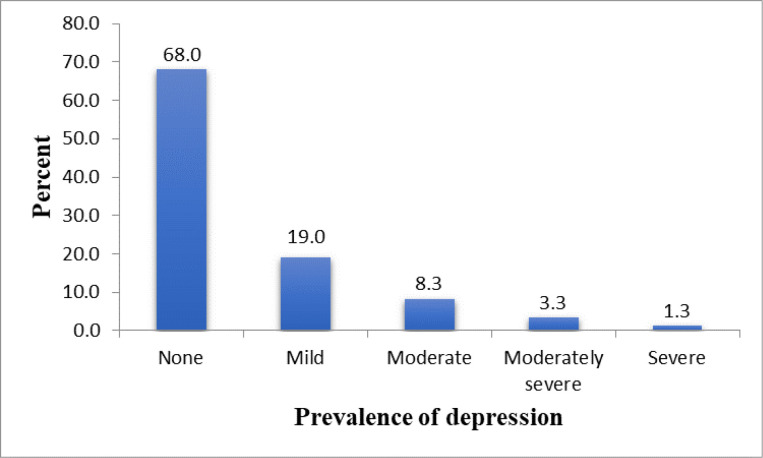
Prevalence of depression among the medical students

Figure 1 shows the prevalence of depression among the students. Overall, 96 (32.0%) were found to have features ranging from mild through moderate to severe depression. A higher proportion 59 (19.0%) had mild depression, while only 4 (1.3%) had severe depression. A higher proportion of pre-clinical students, 53 (39.3%) had depression when compared to the clinical students, 43 (26.1%).

So many factors which are potential risk of developing depression were identified among the students studied (Table 3). These include academic activities such as failing an examination (64.7%); major life events such as loss of a friend (51.3%), loss of a family member (49.7%), physical abuse (14.3%) and sexual abuse (12.3%); emotional problems (57.3%); medical problems such as surgery (21.3%) and chronic medical illness (5.0%); economic problems such as financial challenges (48.0%); and social problems such as alcoholism (11.7%) and smoking (8.0%).

**Table 3:** Identified risk factors of depression among the medical students (n = 300)*

Variables	n (%)
**Failed an examination**	194 (64.7)
**Emotional problems**	172 (57.3)
**Loss of a friend**	154 (51.3)
**Loss of a family member**	149 (49.7)
**Financial challenges**	144 (48.0)
**Serious illness of surgery**	64 (21.3)
**Physical abuse**	43 (14.3)
**Sexual abuse**	37 (12.3)
**Alcoholism**	35 (11.7)
**Smoking**	24 (8.0)
**Unplanned pregnancy**	16 (5.3)
**Chronic medical illness**	15 (5.0)
**Trouble with school authorities**	15 (5.0)
**Trouble with the law**	7 (2.3)
**Taking hard drugs**	7 (2.3)
**Trouble with the law**	7 (2.3)

* Multiple responses

The factors associated with depression among the medical students are shown in Table 4. The bivariate analysis showed that age group (p = 0.009), level of study (p = 0.015), emotional problems (p < 0.001), financial challenges (p < 0.001), physical abuse (p = 0.001), sexual abuse (p = 0.007), smoking (p < 0.001), alcoholism (p = 0.003), use of substances of abuse (p = 0.024) and trouble with the law (p = 0.024) were statistically significantly associated with depression.

**Table 4:** Variables associated with depression among the medical students

Variables	Depression		χ^2^	p-value
	Yes, n (%)	No, n (%)		
**Age group** **(years)**				
**15 – 19**	36 (44.4)	45 (55.6)	11.7	**0.009** *
**20 – 24**	48 (28.9)	118 (71.1)		
**25 – 29**	12 (27.9)	31 (72.1)		
**30 – 34**	0 (0.0)	10 (100.0)		

**Sex**				
**Male**	49 (32.4)	92 (65.2)	0 .926	0.336
**Female**	47 (20.0)	112 (70.4)		

**Marital status**				
**Single**	94 (32.4)	196 (67.6)		
**Married**	2 (20.0)	8 (80.0)	0.685	0.408

**Level of study**			
**Pre-clinical**	53 (39.3)	82 (60.7)		
**Clinical**	43 (26.1)	122 (73.9)	5.994	**0.015** *

**Residence**				
**On campus**	68 (31.1)	151 (68.9)	0.336	0.562
**Off campus**	28 (34.6)	53 (65.4)		

**Reside alone**				
**Yes**	23 (30.3)	53 (69.7)	0.141	0.707
**No**	73 (32.6)	151 (67.4)		

**Emotional** **problems**				
**Yes**	41 (53.9)	35 (46.1)	22.532	**<0.001** *
**No**	55 (24.6)	169 (75.4)	39.706	

**Financial** **challenges**				
**Yes**	56 (56.0)	44 (44.0)	10.651	**<0.001** *
**No**	40 (20.0)	160 (80.0)		

**Physical** **abuse**				
**Yes**	23 (53.5)	20 (46.5)	7.263	**0.001** *
**No**	73 (28.4)	184 (71.6)		

**Sexual abuse**				**0.007** *
**Yes**	19 (51.4)	18 (48.6)	26.671	
**No**	77 (29.3)	186 (70.7)		

**Smoking**				
**Yes**	19 (79.2)	5 (20.8)	9.044	**<0.001** *
**No**	77 (27.9)	199 (72.1)		

**Alcoholism**				
**Yes**	19 (54.3)	16 (45.7)	5.121	**0.003** *
**No**	77 (29.1)	188 (70.9)		

**Use of substance of** **abuse**				
**Yes**	5 (71.4)	2 (28.6)	5.121	**0.024** *
**No**	91 (31.1)	202 (68.9)		

**Trouble with** **the law**				**0.024** *
**Yes**	5 (71.4)	2 (28.6)		
**No**	91 (31.1)	202 (68.9)		

* Statistically significant

χ^2^=Chi square.

All variables with p-value ≤ 0.2 in the bivariate analysis were fitted into the binary logistic regression model to identify independent predictors of depression among the medical students (Table 5). The model revealed that emotional problems (OR 2.205, 95% CI 1.122 – 3.749), financial challenges (OR 3.971, 95% CI 2.170 – 7.269) and smoking (OR 6.877, 95% CI 1.731 – 27.327) were significant independent predictors of depression.

**Table 5:** Logistic regression model for independent predictors of depression among the medical students

Variables	Regression coefficient	Adjusted Odds Ratio (AOR)	95% CI for AOR Lower Upper	p-value
**Age group** **(Years)**	21.780	2878271238.2	0.000	0.999
**15 – 19**	21.054	1391851165.6	-	0.999
**20 - 24**	20.856	1141408996.4	0.000	0.999
**25 - 29**		1	-	
**30 - 34** *			0.000	

**Level of study**				

**Pre-clinical**	0.032	1.032	0.483 2.205	0.935

**Clinical** *		1		
**Emotional** **problems**				

**Yes**	0.718	2.051	1.122 3.749	**0.020**

**No** *		1		

**Financial** **challenges**				

**Yes**	1.379	3.971	2.170 7.269	**<0.001**

**No** *		1		
**Sexual** **abuse**				
**Yes**	0.493	1.637	0.716 3.740	0.243

**No** *		1		

**Smoking**				

**Yes**	1.928	6.877	1.731 27.327	**0.006**

**No** *		1		

**Alcoholism**				

**Yes**	-0.569	0.566	0.180 1.780	0 . 3 30

**No** *		1		

**Use of substance** **of** **abuse**				

**Yes**	1.055	2.873	0.149 55.514	0.485

**No** *		1		

**Trouble** **with the** **law**				

**Yes**	-0.821	0.440	0.052 3.691	0.449

**No** *		1		
**Constant**	-23.072			

* Reference category

## Discussion

This study revealed a high prevalence of depression ranging from mild to severe forms and an avalanche of risk factors which could predispose students to depression. Emotional problems, financial challenges and smoking were the significant independent predictors of depression.

This high prevalence portrays a significant problem with the psychological well-being of medical students, mainly adolescents and young adults. The WHO asserts that “globally, depression is one of the leading causes of illness and disability among adolescents”.^26^ This study has unveiled a huge population of medical students in dire need of mental health promotion and prevention interventions.

The prevalence rate of depression in this study is comparable to what has been reported in studies in South West and South Eastern Nigeria,^8^–^10^ Cameroon; 30.6%^6^, Nepal; 29.78%,^27^ Malaysia; 32.5% and 35.9%,^21^,^28^ India; 31%,^29^ Saudi Arabia; 28.3%,^19^ and Brazil; 30.6%,^5^ A study in Ethiopia reported a much higher prevalence of 51.3%.^7^ These global findings showed that depression is an alarming endemic among undergraduate medical students. This underscores the need to include thorough counselling and preventive mental health services into the routine medical examinations for new students admitted into medical schools. This will lead to early detection of students at risk of developing depression so that care and supported activities can be initiated early enough to prevent future complications such as increased morbidity, suicidal ideation and mortality due to suicide. Recently, there has been a rising incidence of suicide among university students in Nigeria.

In this study, apart from the age group and level of study, other socio-demographic characteristics did not show statistical significance with the prevalence of depression, even though a slightly higher prevalence was recorded among the male students. This finding is consistent with several other studies that have documented that younger age groups are more prone to depression but contrasted the fact that females were significantly more associated with depression.^5^–^7^,^11^,^27^,^28^,^30^ The pre-clinical students were also found to have a higher risk of depression. This could be explained by the fact that the pre-clinical level may probably be associated with fear and anxiety among students because of the structure of the medical curriculum. The introduction to unfamiliar subjects like anatomy, which involves cadaver dissection, could be a source of stress. There may also be fear of failing the examination at the end of the pre-clinical stage, which qualifies students to proceed to the clinical stage. The University regulation stipulates automatic repeat of the class if students fail any subjects in the first sitting and withdrawal from the medical school if the student fails again while repeating. These can mount undue pressure on students and eventually result in depression. However, good orientation and counselling of students can help students over-come fears, worries and anxiety associated with the pre-clinical level of study. A study in Calabar, Nigeria showed that medical students perceived their training programme as very stressful.^12^

Many of the risk factors for depression identified in this study showed statistically significant association with depression among the students. Although the failure of an examination did not show any statistically significant association with depression, students who failed the examination should be counselled and closely monitored so that they do not lose confidence in themselves, a situation that will predispose them to acts capable of jeopardising their academic pursuit in the university. Studies in Enugu^10^ and Ibadan^31^ Nigeria found a significant association between failing an examination and the prevalence of depression, but in Cameroon^6^ and Vietnam,^32^ there was no significant association between depression and academic performance. The finding of an association between sexual and physical abuse and depression calls for an urgent need to develop and implement sexual harassment policy which is visibly lacking in many Nigerian universities. With proper counselling, victims of physical and sexual abuse can rise above their negative experiences, reducing their predisposition to depression.^33^

This study also found that medical students with emotional problems and financial challenges and those who smoke were at a significantly higher risk of being depressed. Emotional problems resulting from relationships, love and sundry issues abound in young adults such as medical students. Students also face emotional problems from peer pressure, throwing them into inner conflict on whether to engage in some activities, which could lead to depression.^13^,^14^ Also, students may decide to take to smoking, alcohol consumption and substance abuse to deal with stress, emotional and other problems that they may face during their studies.^14^ Apart from the physical health effects of smoking, which is a precursor to the use of hard drugs and substance abuse, there is also a psychological effect, with depression featuring very prominently.^15^–^17^ Therefore, the university authority should institute smoking and substance abuse prevention measures as this will go a long way in improving the quality of their lives and reducing their risk of developing depression and other mental health disorders.

Our study has the following limitations: The assessment of depression was done using a screening tool (PHQ-9) based on two weeks prior to the study. Self-reporting by the students may have been prone to information bias. Also, students with severe depression may not be able to answer the questions appropriately. Finally, the results of this study may not be generalized to all medical students in Nigeria since it was carried out in only one university. This underscores the need for a larger multi-centre study involving many medical schools in Nigeria.

## Conclusion

The prevalence of depression was high among medical students. Students with emotional problems, financial challenges and smokers were at a significantly higher risk of developing depression. We recommend that the university authority include screening for risk factors of depression in the routine medical examinations of new students admitted into medical school.
